# Gudgeon fish with and without genetically determined countershading coexist in heterogeneous littoral environments of an ancient lake

**DOI:** 10.1002/ece3.8050

**Published:** 2021-08-31

**Authors:** Tomoyuki Kokita, Kohtaro Ueno, Yo Y. Yamasaki, Masanari Matsuda, Ryoichi Tabata, Atsushi J. Nagano, Tappei Mishina, Katsutoshi Watanabe

**Affiliations:** ^1^ Faculty of Marine Science and Technology Fukui Prefectural University Obama Japan; ^2^ National Institute of Genetics Mishima Japan; ^3^ Lake Biwa Museum Kusatsu Japan; ^4^ Faculty of Agriculture Ryukoku University Otsu Japan; ^5^ Institute for Advanced Biosciences Keio University Tsuruoka Japan; ^6^ Laboratory for Chromosome Segregation RIKEN Center for Biosystems Dynamics Research Kobe Japan; ^7^ Graduate School of Science Kyoto University Kyoto Japan

**Keywords:** genetic color polymorphism, incipient species, Lake Biwa, melanism, spatially heterogeneous environments

## Abstract

Countershading, characterized by a darker dorsal surface and lighter ventral surface, is common among many animals. This dorsoventral pigment polarity is often thought to be adaptive coloration for camouflage. By contrast, noncountershaded (melanistic) morphs often occur within a species due to genetic color polymorphism in terrestrial animals. However, the polymorphism with either countershaded or melanistic morphs is poorly known in wild aquatic animals. This study explored the genetic nature of diverged color morphs of a lineage of gudgeon fish (genus *Sarcocheilichthys*) in the ancient Lake Biwa and propose this system as a novel model for testing hypotheses of functional aspects of countershading and its loss in aquatic environments. This system harbors two color morphs that have been treated taxonomically as separate species; *Sarcocheilichthys variegatus microoculus* which occurs throughout the littoral zone and *Sarcocheilichthys biwaensis* which occurs in and around rocky areas. First, we confirmed that the divergence of dorsoventral color patterns between the two morphs is under strict genetic control at the levels of chromatophore distribution and melanin‐related gene expression under common garden rearing. The former morph displayed sharp countershading coloration, whereas the latter morph exhibited a strong tendency toward its loss. The crossing results indicated that this divergence was likely controlled by a single locus in a two‐allele Mendelian inheritance pattern. Furthermore, our population genomic and genome‐wide association study analyses detected no genome‐wide divergence between the two morphs, except for one region near a locus that may be associated with the color divergence. Thus, these morphs are either in a state of intraspecific color polymorphism or two incipient species. Evolutionary forces underlying this polymorphism appear to be associated with heterogeneous littoral environments in this lake. Future ecological genomic research will provide insight into adaptive functions of this widespread coloration, including the eco‐evolutionary drivers of its loss, in the aquatic world.

## Graphical abstract

Divergent color phenotypes with or without countershading in *Sarcocheilichthys* inhabiting Lake Biwa, Japan. This study confirmed the coexistence of genetically determined color morphs with and without countershading for the *Sarcocheilichthys* system. The two morphs are a state of genetic color polymorphism within a single interbreeding population or two incipient species potentially maintained by heterogeneous littoral environments in this ancient lake.

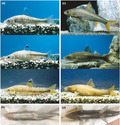

## 1 INTRODUCTION

The adaptive functions of animal coloration are a widespread and fascinating topic in ecology and evolutionary biology (Cuthill et al., 2017). Countershading, characterized by a light ventral surface and darker dorsal surface, is among the most common color patterns in terrestrial and aquatic animals including mammals, birds, reptiles, fishes, and insects (Kiltie, 1988; Thayer, 1896). This dorsoventral pigment polarity is ecologically explained as an adaptation for camouflage through the self‐shadow concealment and/or background matching, although it may have other benefits such as enhancing thermoregulation or protection against ultraviolet light (reviewed by Rowland, 2009; Ruxton et al., 2004). By contrast, noncountershaded (uniformly dark‐colored) phenotypes, which are associated with increased melanin pigmentation on the ventral surface, known as melanism, are also widespread among terrestrial animals (Allen et al., 2012; Brown et al., 2017; Caro, 2005). Both genetically determined countershading and melanistic morphs can occur within a single population, and morph frequency variation has been associated with environmental heterogeneity (e.g., Nachman, 2005; Potash et al., 2020). Such color polymorphisms often have a simpler genetic basis than other ecologically relevant traits (McKinnon & Pierotti, 2010; Orteu & Jiggins, 2020). Therefore, they provide excellent opportunities for understanding the adaptive significance of animal coloration and elucidating the range of evolutionary processes that contribute to the maintaining genetic polymorphisms within and between populations (Gray & McKinnon, 2007; White & Kemp, 2016).

Many aquatic organisms are also countershaded, and the concealing function of countershading may be more beneficial underwater than in terrestrial habitats (Kiltie, 1988; Rowland, 2009; Ruxton et al., 2004). Thus, countershading is thought to be a more effective antipredator adaptation among aquatic prey species, where predators can be attacked from all directions, because dark dorsal pigmentation provides a good match to the dark background when the prey is viewed from above, whereas a light ventral surface would match the downwelling light from overhead when it is viewed from below. Partly for this reason, the loss of countershading is far less common in aquatic habitats than in terrestrial systems (Venables et al., 2019). However, the hypotheses about the ecological and evolutionary mechanisms of using countershading to attain crypsis remain poorly tested in wild aquatic organisms (Kelley & Merilaita, 2015; Kelley et al., 2017). Although some examples of noncountershaded coloration have been reported, the ecological drivers and evolutionary genetic basis underlying dark pigmentation remain unknown (Caro et al., 2011; Lindgren et al., 2014; Venables et al., 2019). A productive way to address these questions including the functional aspects of such color patterns would be to examine aquatic animals that exhibit stable color polymorphism with either countershaded or melanistic morphs and are amenable to ecological and genetic analyses.

**FIGURE 1:**
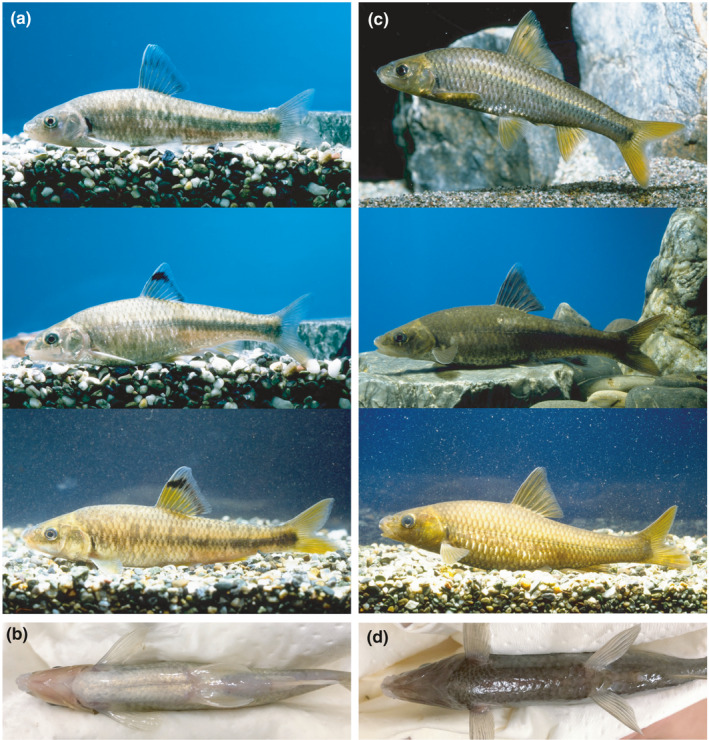
Divergent color phenotypes with or without countershading in *Sarcocheilichthys* inhabiting Lake Biwa, Japan. Photographs of a countershaded morph taxonomically described as *S*. *variegatus microoculus* taken from the side (a) and below (b). Photographs of a melanistic morph taxonomically described as *S*. *biwaensis* taken from the side (c) and below (d). A variety of body color patterns, especially in terms of yellow coloration, were observed for each morph. Photographs in (a) and (c) are provided courtesy of the Lake Biwa Museum

As a potential candidate for this, we focused on a lineage of the gudgeon fish (genus *Sarcocheilichthys*, belonging to the subfamily Gobioninae, the family Cyprinidae), which inhabit the littoral zones of Lake Biwa, an ancient temperate lake in central Japan. Lake Biwa (surface area 670 km^2^; maximum depth 104 m) is the oldest lake in East Asia dating to about 4 million years ago (Kawabe, 1994; Yokoyama, 1984), and its littoral zone comprises complicated bottom environments including sandy, pebbly, and rocky areas (see Komiya et al., 2011). The lake harbors 16 endemic fish species/subspecies (hereafter species) including two *Sarcocheilichthys* species (reviewed by Okuda et al., 2014). These two species have divergent body colors which are not sex‐linked and distributional patterns (Hosoya, 1982; Nakamura, 1969). *Sarcocheilichthys variegatus microoculus* has a yellow‐grayish body and occurs throughout the littoral zone and in rivers flowing into Lake Biwa, whereas *Sarcocheilichthys biwaensis* has a brownish body with yellow‐brownish and brownish‐black coloration, and occurs strictly in and around rocky areas. Thus, the two species co‐occur in rocky areas, showing spatial variation in their frequency, and *S*. *biwaensis* could be found up to about 30% in these areas (Nakamura, 1969). At least visually, the former species display typical countershading and the latter species seems to exhibit dark pigmentation over its entire body including the ventral region and lack countershading pattern (see Figure 1). Although these fishes have been treated taxonomically as separate species, previous phylogeographic and population genetic analyses using neutral DNA markers have suggested no genetic differentiation between these species and the panmictic status of *Sarcocheilichthys* in Lake Biwa (Komiya et al., 2011, 2014; Tabata et al., 2016). Therefore, we propose this *Sarcocheilichthys* system as a model of stable color polymorphism, with countershaded and melanistic morphs. However, to date, no study has examined the color pattern divergence or genetic aspects of color polymorphism in this system in detail, except for Nakamura (1969)'s brief description (data not shown).

Here we assessed the following questions in order to establish the *Sarcocheilichthys* system as a novel model for studying the evolutionary mechanisms of countershading color polymorphism in the aquatic world. First, we examined whether the divergence of dorsoventral color patterns in this system is under strict genetic control at the levels of chromatophore distribution and melanin‐related gene expression. Second, we further examined whether this phenomenon is controlled by a few genes exhibiting Mendelian segregation, as has been shown in other taxa. Last, we applied restriction site‐associated DNA sequencing (RAD‐seq) to explore the nature of genome‐wide pattern of genetic divergence between these color morphs (i.e., taxonomic species) in an area where they coexist, to obtain evidence of color polymorphism within a single interbreeding population for this system.

## 2 MATERIALS AND METHODS

### 2.1 Crossing experiment and common garden rearing

As mentioned above, regardless of their taxonomic status, the *Sarcocheilichthys* system in Lake Biwa appears to represent color polymorphism within a single population or a pair of incipient species with weak state of premating isolation (see also Discussion). Therefore, for convenience, we will refer to *S*. *variegatus microoculus* as the S_V_ morph and *S*. *biwaensis* as the S_B_ morph. Intra‐ and intermorph crossing of the *Sarcocheilichthys* system can be achieved through artificial insemination using mature wild individuals (Nakamura, 1969). Therefore, to create experimental families for common garden rearing, backcrossing, and F_2_ hybrid populations, we collected parental fish (three males and three females) of the S_V_ morph from a tributary of the Ane River, which flows into Lake Biwa, and parental fish (three males and two females) of the S_B_ morph around off Onoe in Lake Biwa. These sampling locations are indicated in Komiya et al. (2014) as locality codes 9 and 7, respectively. The parental individuals were transferred to 180‐L tanks, where they were maintained until artificial insemination.

**FIGURE 2:**
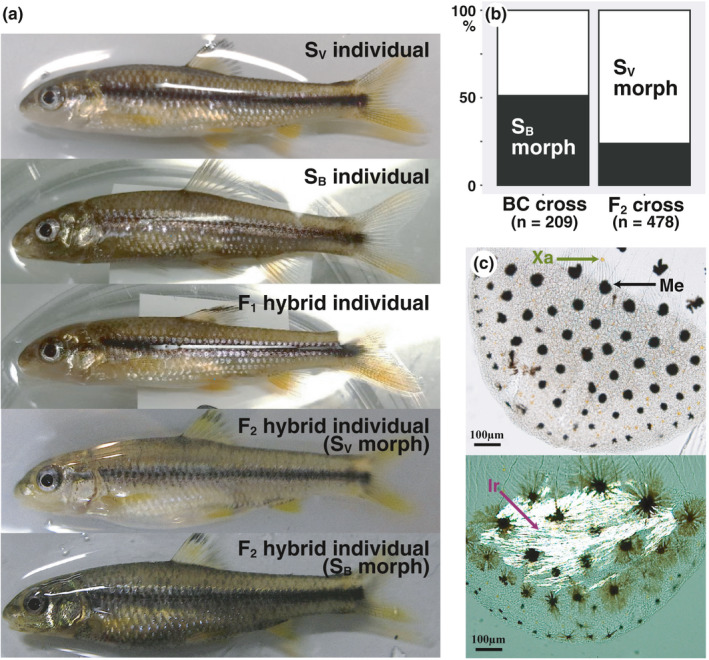
Color phenotypes of a pure cross of the *S*. *variegatus microoculus* (S_V_) morph, a pure cross of the *S*. *biwaensis* (S_B_) morph, an F_1_ hybrid cross, and an F_2_ hybrid population between the two morphs under common garden rearing (a). The frequencies of individuals expressing either S_V_ or S_B_ color phenotypes in a backcrossed (BC) population and an F_2_ intercross population, respectively (b). Chromatophores (Me: melanophore; Xa: xanthophore; Ir: iridophore) on the scales of *Sarcocheilichthys* fish (c)

We created two full‐sibling families of the S_V_ morph (F_V1_ and F_V2_), two full‐sibling families of the S_B_ morph (F_B1_ and F_B2_), and a hybrid family (F_H1_) between the two morphs. We also created a mixed family (F_BM_) from fertilized eggs of the S_B_ morph from captive broodstock maintained at the Lake Biwa Museum (Japan). Artificial insemination was performed from May to June 2015 using eggs from each ovulated female and sperm from each male, which were obtained by pushing the belly. An F_1_ intercross family (F_H1_) was created using a male S_V_ morph that was not used for creation of full‐sibling families and a female S_B_ morph that was also used to create F_B2_. The fertilized eggs were transferred into a Petri dish (φ152 mm) filled with fresh water and kept at 20°C. After hatching, the progeny (about 30 individuals per family) were reared in a 40‐L tank under natural light conditions at 20–25°C. The progeny were fed live *Artemia* nauplii, frozen *Daphnia,* and frozen bloodworms (*Chironomus* spp.), depending on their size, ad libitum. Individuals that reached about 40 mm in total length were sacrificed for chromatophore and gene expression analyses (Figure 2a).

To determine the inheritance pattern of divergent coloration, we generated another F_1_ intercross family (F_H2_) between a male S_B_ morph and a female S_V_ morph that were not used in previous crosses, in May 2014. After hatching, the progeny were reared until maturity in a 180‐L tank. From May to June 2016, we created a backcross (BC) population from artificially fertilized eggs using sperm from the father individual and eggs from 18 mature hybrid females and an F_2_ hybrid population by crossing one mature F_1_ hybrid male and 18 F_1_ hybrid females. The progeny were reared in 60‐L tanks (about 50 individuals per tank) under the light, temperature, and feeding conditions described above until they reached about 40 mm in total length, and then their color phenotypes were visually observed. The BC and F_2_ populations comprised 209 and 478 individuals, respectively (Figure 2b).

### 2.2 Chromatophore quantification and melanin‐related gene expression

**FIGURE 3:**
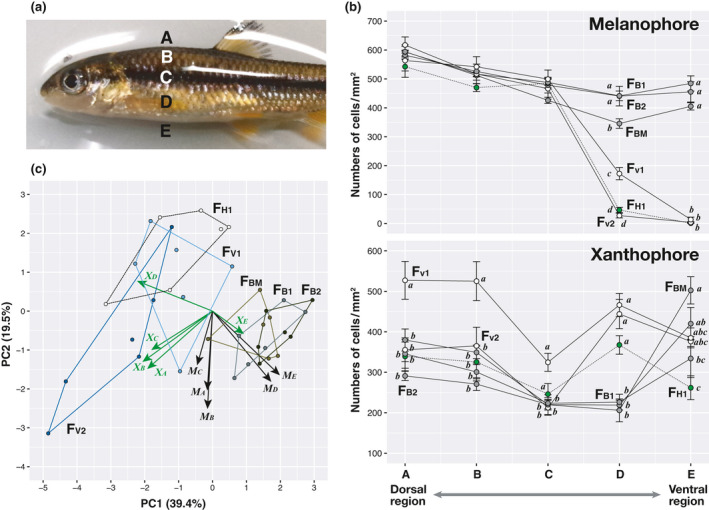
Comparison of dorsoventral chromatophore distribution patterns between the *S*. *variegatus microoculus* (S_V_) and *S*. *biwaensis* (S_B_) morphs. Five selected regions (A–E) for chromatophore counts are shown (a). The mean (and SE) cell numbers (per mm^2^ on scale skin) of melanophores and xanthophores for each region in each family (*n* = 6 individuals per family) were used for this analysis (b). Tukey–Kramer multiple comparison test results are provided for each region. Symbols with the same letters were not significantly different among families (*p* < 0.05). Melanophore density was not significantly different in regions A–C, according to all pairwise comparisons. Scatterplot of the first two axes derived from PCA of chromatophore data (c). Percentage of variance explained by the axes is given in parentheses. Convex hulls for families and vectors for variables (M and X: melanophore and xanthophore densities for each region, respectively) are also drawn

We counted the individual chromatophores on the scales of each morph and compared their dorsoventral distribution throughout the body. Like the zebrafish (*Danio rerio*), which is a model cyprinid fish in a wide variety of biological fields (Hirata et al., 2005), *Sarcocheilichthys* chromatophores include melanophores, xanthophores, and iridophores (Figure 2c). We easily detected a dense, uniform sheet of iridophores; however, we did not analyze iridophores in this study because they were difficult to count and less relevant to the objective of this study. To observe melanized melanophores and xanthophores on the scales, six individuals of each experimental family (F_V1_, F_V2_, F_B1_, F_B2,_ F_BM_, and F_H1_) were anesthetized in a 0.05% 2‐phenoxyethanol solution, and scales were collected from five regions of the body between the posterior edge of the head to the anterior edge of the dorsal or anal fin. The selected regions for chromatophore counts include dorsal area around the dorsal midline (A), left side of the body upward lateral line (B), around left lateral line (C), left side of the body downward to the lateral line (D), and belly around the ventral midline (E) (Figure 3a). We immersed 10 scales in phosphate‐buffered saline (pH 7.2) containing 2 mg/ml epinephrine (Sigma‐Aldrich) for 60 min to contract melanosomes. Photomicrographs of each scale were taken using a microscope (Eclipse 80i, Nikon) equipped with a digital camera. The melanophores and xanthophores on each scale were counted, and their densities (per mm^2^) in the scale skin were calculated using the WinROOF software ver. 5.6 (Mitani Co.). Data for each region in each individual are means of 10 scales.

To confirm divergence in the expression patterns of melanin‐related genes between the two morphs, we selected two important genes regulating melanin production, tyrosinase (TYR) gene and dopachrome tautomerase (DCT). DCT is conserved as a single‐copy gene across teleost genomes (Braasch et al., 2007). Although TYR gene is also present as a single copy in the zebrafish genome, many teleosts have two copies of this gene (Braasch et al., 2007). Therefore, at this time, the possibility that *Sarcocheilichthys* fishes have two TYR copies cannot be ruled out completely. The elongation factor 1 alpha (EF1α) gene was used as an internal control. EF1α gene has stable expression level during development and across tissue types, and is recommended as housekeeping gene in zebrafish (McCurley & Callard, 2008).

We used six individuals from each experimental family (excluding F_BM_) for the reverse transcription‐quantitative PCR (RT‐qPCR) analysis. Total RNA was extracted from 10 scales on the dorsal (A) and ventral (E) areas of each individual using the RNeasy Micro Kit (Qiagen). Genomic DNA was digested using the RNase‐Free DNase Set (Qiagen). cDNA was synthesized from extracted RNA (100 ng) using the High‐Capacity cDNA Reverse Transcription Kit (Life Technologies). To design primers for RT‐qPCR, the partial coding regions of TYR, DCT, and EF1α genes from each morph were sequenced using cDNA from scale RNA. We searched for a highly conserved region of each gene across cyprinid fishes deposited in the public DNA database and designed primers to amplify the partial coding regions of these genes. The PCR primers were as follows for TYR (forward 5′‐GACTTTAACTTCACCATCCCGTA‐3′ and reverse 5′‐CCTGCACAAACCGCTGAC‐3′), DCT (forward 5′‐TAACTGTGGCGAGTGTAAGTTCG‐3′ and reverse 5′‐TGGTTGGTAAAGTCACATTAGTGC‐3′) and EF1α (forward 5′‐TGATCTACAAATGCGGTGGA‐3′ and reverse 5′‐CATCCTGAAGTGGCAGACG‐3′). Direct sequencing from the PCR products indicated that no individual had multiple peaks.

The real‐time quantitative PCR was conducted using the CFX Connect Real‐Time PCR Detection System with the SsoAdvanced SYBR Green Supermix (Bio‐Rad). Six individuals from each family (excluding F_BM_) were used in this analysis. The primers of TYR (forward 5′‐AGGAAACCATGACCGAACC‐3′ and reverse 5′‐CTCGCAAACCCTTCCAGAG‐3′; product size 144 bp), DCT (forward 5′‐TCGTCTGGCAGCACTACTACTC‐3′ and reverse 5′‐CCAGGTTGAGCAGATGGAAG‐3′; product size 129 bp) and EF1α (forward 5′‐AACGGACAGACCCGTGAAC‐3′ and reverse 5′‐TGTAGGCGCTGACTTCCTTG‐3′; product size 145 bp) were designed using sequencing data from the partial coding regions of these genes. The PCR protocol was 3 min at 95°C followed by 40 cycles of 5 s at 95°C, 30 s at 60°C. The specificity of the reaction was verified through melting curve analysis and electrophoresis of the amplified PCR products. All expression assays were performed in duplicate. The relative expression levels were determined by the comparative (ΔΔCt) method (Livak & Schmittgen, 2001) after similar amplification efficiencies between the target and reference genes (EF1α) were validated. The threshold cycle (Ct) value of EF1α did not differ significantly by family or scale region (two‐way ANOVA, family: *F*_4,50_ = 1.46, *p* > 0.22; scale region: *F*_1,50_ = 1.28, *p* > 0.26; interaction: *F*_4,50_ = 2.03, *p* > 0.10).

All statistical analyses were performed using R version 4.0.2 (R Core Team, 2020). Significance was determined at a level of *p* < 0.05.

### 2.3 Population genomic and genome‐wide association study (GWAS) analyses

To assess the degree of genome‐wide divergence between the two morphs, we performed population genomic analysis of coexisting population using double‐digest RAD‐seq (ddRAD‐seq; Peterson et al., 2012). Sixteen individuals of each morph were collected around off Onoe in Lake Biwa (see above). This site has been known as the co‐occurring area of both morphs (Hosoya, 1982; Komiya et al., 2011). Most of these individuals were used in previous studies (Komiya et al., 2011, 2014). Genomic DNA was extracted from the pectoral or caudal fins using DNeasy Blood & Tissue Kit (Qiagen). A ddRAD library was prepared according to Peterson's protocol with slight modifications (Sakaguchi et al., 2015). In this study, genomic DNA was digested using MseI and BglII (New England Biolabs) and the library size was selected into 350‐ to 550‐bp fragments by running on a 2% agarose gel. The samples were sequenced on the Illumina HiSeq X Ten with 150 bp paired‐end reads.

The RAD‐seq reads were filtered using fastp v.0.20.1 to remove the Illumina adapter and to eliminate low‐quality regions (parameters used: ‐‐adapter_sequence = AGATCGGAAGAGCACACGTCTGAACTCCAGTCA ‐‐adapter_sequence_r2 = AGATCGGAAGAGCGTCGTGTAGGGAAAGAGTGT ‐‐cut_right ‐‐cut_window_size 4 ‐‐cut_mean_quality 20 ‐l 100 ‐w 1 ‐‐max_len1 100 ‐‐max_len2 100) (Chen et al., 2018). *De novo* assemble of RAD sites and SNP calling was performed using the *denovo_map.pl* pipeline of Stacks v.2.55 (Rochette et al., 2019) with the parameters: ‐n 3 ‐M 3 ‐‐paired. The *populations* program of Stacks was used to output and filter SNPs (parameters used: ‐p 2 ‐r 0.6 ‐R 0.6 ‐‐max‐obs‐het 0.7 ‐‐min‐maf 0.05 ‐‐write‐single‐snp). Finally, 41,804 SNPs were obtained and used in subsequent population genetic analyses.

Clustering analysis was performed by ADMIXTURE v.1.3.0 (Alexander et al., 2009). ADMIXTURE was run with *K* assumption ranging from 1 to 4. The results were summarized and graphically displayed using CLUMPAK (Kopelman et al., 2015). Cross‐validation (CV) scores were used to determine the best *K* value. Next, the principal component analysis (PCA) was performed using the R package *adegenet* v.2.1.3 (Jombart, 2008; Jombart & Ahmed, 2011). We also calculated *F*_ST_ value between the two morphs by GenoDive v.3.04 (Meirmans, 2020). Significance tests were conducted by 10,000 rounds of permutations.

In addition, a GWAS was conducted to define the genetic determinant of the two color phenotypes using the ddRAD SNP dataset. We used the method genome‐wide efficient mixed‐model association (GEMMA) (Zhou & Stephens, 2012), which calculates statistical genotype–phenotype associations implementing the GEMMA v.0.97. Color phenotype was treated as the binary variable, and a restricted set of 10,950 SNPs with missingness below 5% (default setting of GEMMA) were used for this analysis. A univariate linear mixed model was applied to account for relatedness among individuals. *P*‐values obtained from the Wald test were corrected for multiple testing (Bonferroni method), and SNPs with corrected *p*‐values lower than 0.05 were retained for possible association with color phenotype.

## 3 RESULTS

### 3.1 Genetic crosses

All individuals within S_V_ families (*n* = 26 for F_V1_; *n* = 30 for F_V2_) exhibited countershading, whereas those of S_B_ families (*n* = 21 for F_B1_; *n* = 30 for F_B2_; *n* = 28 for F_BM_) did not, as evaluated by appearance alone (Figure 2a). In each of these families, the number of melanophores tends to decrease from the dorsal to ventral regions (one‐way repeated‐measures ANOVA, *F*_4,20_ = 5.1–219.7, *p* < 0.006; Figure 3b). Although it did not differ significantly for A to C regions between the S_V_ and S_B_ morphs (nested ANOVA where family was nested within morphs, *F*_1,3_ = 0.001–1.15, *p* > 0.36), the latter morph had a greatly higher number for D and E regions than the former morph, which nearly lack melanophores in ventral regions (*F*_1,3_ = 20.8, *p* < 0.02 for D, *F*_1,3_ = 213.2, *p* < 0.0001 for E). Although xanthophore numbers differed significantly among regions within each family (one‐way repeated‐measures ANOVA, *F*_4,20_ = 3.9–28.8, *p* < 0.018), we observed neither a clear dorsoventral pattern nor a difference between morphs in the xanthophore numbers (Figure 3b). In addition, there was no significant difference between the two morphs except for D region (nested ANOVA where family was nested within morphs, *F*_1,3_ = 0.4–4.3, *p* > 0.12 for A, B, C, and E; *F*_1,3_ = 446.5, *p* < 0.0003 for D). These chromatophore distribution patterns were supported by the PCA results (Figure 3c); PC1 explained 39.4% of the total variance, easily distinguishing the two morphs.

**FIGURE 4:**
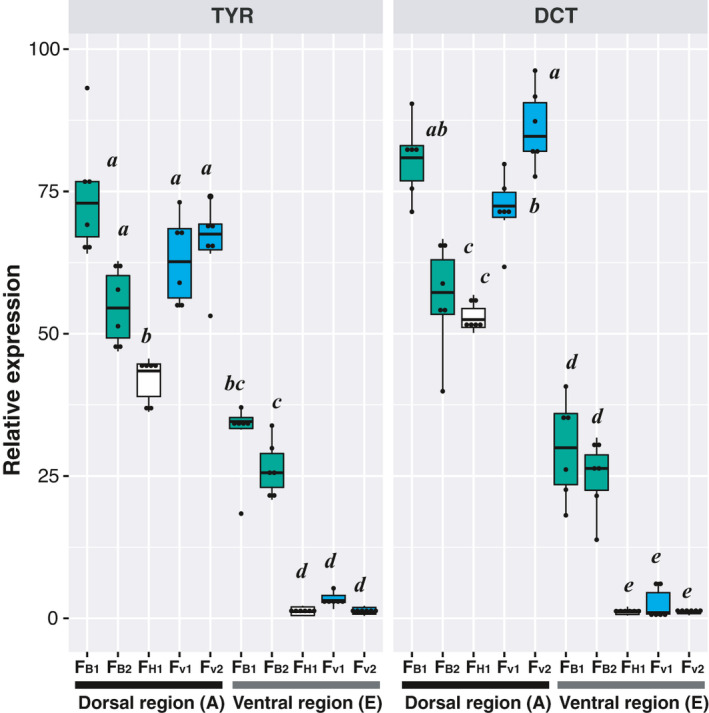
Comparison of dorsoventral expression levels of two melanin‐related genes (TYR and DCT) between five families of the two color morphs. Two regions of scale skin (A and E in Figure 3a) were selected for RT‐qPCR. The lower and upper limits of each box correspond to the first and third quartiles, and the horizontal line within each box indicates the median. Whiskers extend to the lowest and highest observed biases within 1.5 interquartile range units from the box. For each gene, results of the Tukey‐Kramer multiple comparison test (following one‐way ANOVA) are provided. Symbols with the same letters indicate no significant difference (*p* < 0.05)

The qPCR results for the two melanin‐related genes indicated higher expression levels in dorsal regions than in ventral regions in all families, with exceptionally higher expression levels in the ventral region in S_B_ families (F_B1_ and F_B2_) than in S_V_ families (F_V1_ and F_V2_) (one‐way ANOVA, TYR: *F*_7,40_ = 114.7, *p* < 0.0001; DCT: *F*_7,40_ = 162.0, *p* < 0.0001; Figure 4). Thus, the S_V_ morph showed a sharp countershading pattern, whereas the S_B_ morph exhibited a strong (but not complete) tendency losing dorsoventral countershading at the level of chromatophore distribution and gene expression which regulates melanin production.

The offspring color ratios of hybrid crosses indicated that color divergence between these morphs was likely controlled by a single locus, that is, a two‐allele Mendelian inheritance pattern. All progeny of the two F_1_ hybrid families (*n* = 29 for F_H1_; *n* = 32 for F_H2_) exhibited countershading (see Figure 2a). Patterns in the overall distribution of melanophores and melanin‐related gene expression were almost identical between the F_H1_ family and S_V_ families (F_V1_ and F_V2_), suggesting that the allele for countershading is dominant (Figures 3, 4). In a backcrossed population, 102 and 107 individuals showed countershaded and melanistic color phenotypes, respectively, which was not significantly different from the expected 1:1 ratio (two‐tailed exact binomial test, *p* > 0.78; Figure 2b). In an F_2_ intercross population, 364 and 114 individuals showed countershaded and melanistic color phenotypes, respectively, which was not significantly different from the expected 3:1 ratio (two‐tailed exact binomial test, *p* > 0.59; Figure 2b).

### 3.2 Genetic divergence in coexistence populations

**FIGURE 5:**
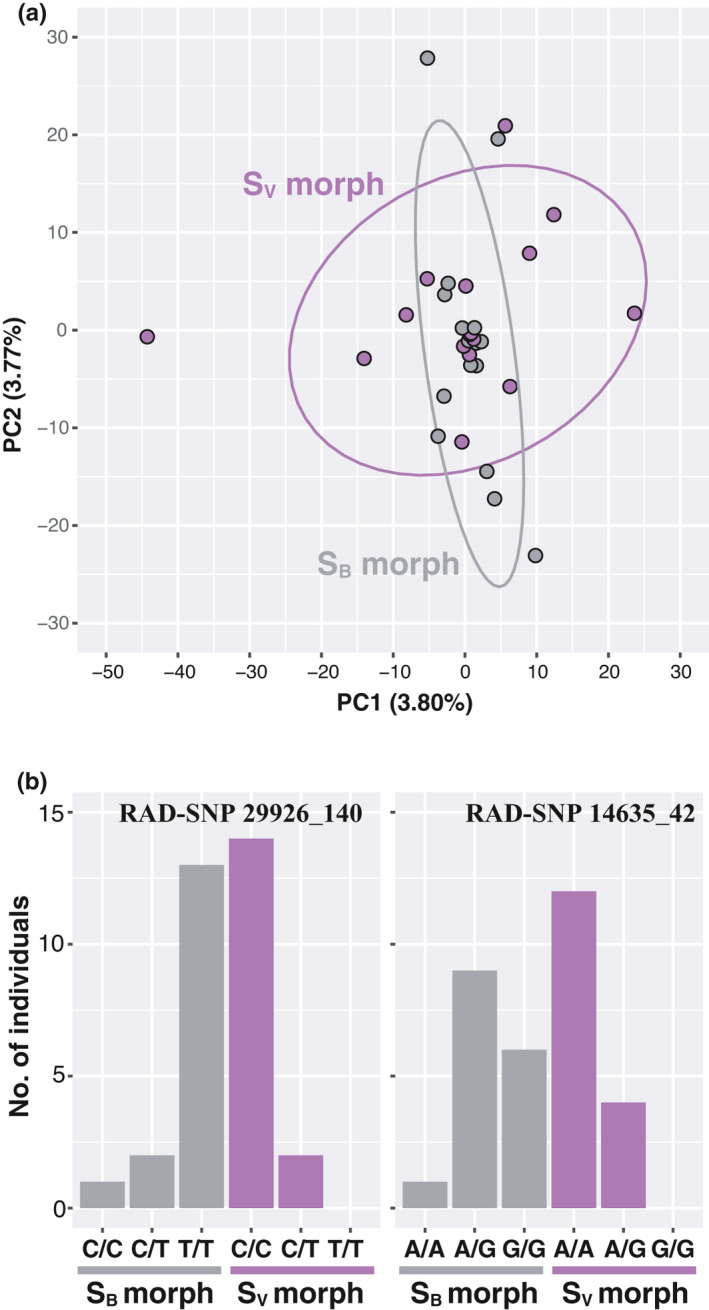
Scatterplot showing individual variation in the first two principal components (percentage of variance in parentheses) derived from PCA conducted on 41,804 SNPs genotyped in 16 individuals each of *S*. *variegatus microoculus* (S_V_) and *S*. *biwaensis* (S_B_) morphs collected in a region where they coexist, with 95% confidence ellipses (a). Frequencies of different genotypes at the two RAD‐SNP sites, 29926_140 and 14645_42, that were found to be significant in GWAS analysis (*p* < 0.05 after Bonferroni correction) in each morph with or without countershading

All of our population genetic analysis results indicated a lack of genome‐wide divergence and population structure between the two morphs in a region where both morphs coexist. In the clustering analysis with ADMIXTURE using 41,804 SNP loci from ddRAD‐seq, *K* = 1 was estimated to be best fit because its CV score was the lowest (Figure S1). The pairwise estimate of genetic differentiation between the two morphs was very low (*F*_ST_ = 0.001) and not significant (*p* = 0.079 after 10,000 permutations). The PCA results were consistent with these findings (Figure 5a). The levels of expected heterozygosity (*H*_E_) and nucleotide diversity (*π*) were almost similar between the two morphs, respectively (*H*_E_ and *π*: 0.286 and 0.296 for S_V_ morph; 0.284 and 0.295 for S_B_ morph).

The GWAS result revealed that each one SNP on two RAD loci 29,926 and 14,635 (*p* = 1.04 × 10^–6^ and *p* = 0.039, respectively, following the Bonferroni correction) was significantly associated with color phenotype, showing an extremely highly significant result for the former locus (Figure 5b, see also Figures S2 and S3). We also detected statistically significant genotypic linkage disequilibrium between the two loci (*p* = 0.0009 by GENEPOP online version 4.7; Raymond & Rousset, 1995).

## 4 DISCUSSION

One objective of this study was to determine whether the diverged color phenotypes of the *Sarcocheilichthys* system in Lake Biwa have a genetic basis. Under common garden rearing, color divergence was clearly shown to be under strict genetic control and not environmentally driven plasticity (e.g., Hochkirch et al., 2008; Roulin, 2014). Although both morphs tended to decrease from the dorsal to ventral regions both at the levels of melanophore distribution and gene expression which regulates melanin production, this dorsoventral gradation of melanin pigmentation on the body differed significantly and showed discontinuity between the two morphs. The S_V_ morph exhibited a near‐total absence of melanized melanophores in the ventral region; conversely, the S_B_ morph is characterized by melanophores occurrence and melanin production throughout the body. Thus, our findings clarified that the S_V_ morph exhibits sharp countershading, whereas the S_B_ morph exhibits a strong (but not complete) tendency toward to lose its dorsoventral countershading. By contrast, xanthophore showed a complex distribution pattern throughout the body and their density differed widely among families of the same morph. For example, individuals of the family F_V1_ had many more xanthophores than the other families. Within this system, we observed considerable, continuous variation in the amount of yellow coloration in each morph (see Figure 1).

Both morphs examined in this study, which are treated as taxonomically separated species that are endemic to Lake Biwa, are presumed to have evolved from the ancestral riverine (sub)species *Sarcocheilichthys variegatus variegatus*, which is widely distributed in western Japan, except in Lake Biwa (Hosoya, 1982). Phylogeographic analyses using neutral DNA markers have suggested that the Lake Biwa population was created by multiple colonizations of this lake by riverine lineages, although a reconsideration of the taxonomic status of these *Sarcocheilichthys* fishes is necessary (see Komiya et al., 2014). Because the ancestral riverine lineages exhibit countershading, at least visually, the melanistic phenotype of the S_B_ morph must have evolved from the countershading phenotype of the ancestral lineages.

The results of our F_1_ hybrid, BC, and F_2_ hybrid crosses indicated that the body color divergence was likely controlled by a single‐locus, two‐allele Mendelian inheritance pattern, and the melanistic allele of the S_B_ morph is recessive, which causes a melanistic phenotype when homozygous. In several mammals and birds, it is known that melanism results from allelic variation in one of two genes, melanocortin receptor 1 (MC1R) and an antagonist for MC1R, agouti‐signaling protein (ASIP), which are involved in the melanocortin system, a complex neuroendocrine signaling mechanism involved in numerous physiological processes including vertebrate pigmentation (e.g., Janssen & Mundy, 2017; Kingsley et al., 2009; McRobie et al., 2019; Nachman et al., 2003; Vidal et al., 2010). In mammals, ASIP binds to MC1R, which is the receptor for *α*‐melanocyte‐stimulating hormone (*α‐*MSH), and binding of ASIP to MC1R lowers the ratio of eumelanin (dark/brown pigment) to pheomelanin (red/yellow pigment) produced in the melanocytes (mammalian melanophores) (reviewed by d'Ischia et al., 2015). Thus, spatially regulated ASIP expression generates the countershaded pattern by driving a switch between the production of chemically distinct melanins in melanocytes in dorsal and ventral regions. Although fish melanophores synthesize only eumelanin, and their cellular and biochemical mechanisms are different from those of mammalian species, dorsoventral gradients of melanin pigmentation in many fishes also appear to depend on a dorsoventral expression gradient of ASIP1, the fish ortholog of mammalian ASIP (reviewed by Cal et al., 2017; Irion & Nüsslein‐Volhard, 2019). A recent study reported that ASIP1 knockout zebrafish created using CRISPR–Cas9 technology exhibited a noncountershaded phenotype similar to melanism (Cal et al., 2019). By contrast, the gene expression of two melanocortin receptors (MC1R and MC5R), but not ASIP1, is higher in darker, more pigmented areas of the Japanese flounder (Matsuda et al., 2018). Each of these genes may be a putative genetic factor involved in color divergence within the *Sarcocheilichthys* system.

Population genetic analyses using 41,804 RAD‐SNP markers indicated no genome‐wide genetic differentiation between the two *Sarcocheilichthys* color morphs. This result strongly supported the findings of a previous study that was based on a restricted dataset (i.e., partial nucleotide sequences of mitochondrial cytochrome *b* gene and 14 microsatellite loci; Komiya et al., 2011). The S_B_ morph has been taxonomically described as an independent species (*S*. *biwaensis*), primarily based on its body (and fin) color, body shape, and head morphology (Hosoya, 1982). Indeed, the S_B_ morph has a longer head and deeper body than its congener. However, the ecomorphological traits of the S_V_ morph (*S*. *variegatus microoculus*), which utilizes several types of bottom environments including sandy, pebbly, and rocky areas, show a remarkable continuous variation in relation to its habitat types, and its head and body shapes in rocky areas largely overlap with those of the S_B_ morph (Komiya et al., 2011). These traits of both morphs inhabiting rocky areas are considered to be advantageous for capturing cryptic and/or attached prey in rocky areas with complex structure (Komiya et al., 2011). Thus, sympatric S_B_ and S_V_ morphs exhibit similar morphologies, except for body coloration, and cannot be discerned by genome‐wide DNA markers, causing the difficult issue of their status.

Generally, the speciation process is continuous, with sympatric divergence occurring along a speciation continuum that ranges from a relatively homogeneous population containing multiple phenotypes (intraspecific polymorphism) to reproductively isolated sister species with clear genetic differentiation (Hendry et al., 2009; Nosil, 2012). Therefore, the panmictic status of the *Sarcocheilichthys* system suggested by genome‐wide analysis lies somewhere along this speciation continuum, between genetic color polymorphism within a single population and development of some reproductive isolation mechanism between color morphs indicating that the isolation is either relatively recent and has not yet accumulated genetic differences (i.e., early stage of speciation or incipient speciation). Although degree of reproductive isolation is typically used as a measure of how far the process of speciation has proceeded, premating isolation between these color morphs, that is, whether or not assortative mating exists between divergent color phenotypes under natural conditions, has not been well examined in the wild. However, we have a limited evidence of weak or lacking premating isolation between the color morphs. In a preliminary artificial crossing experiment using mixed sperm from multiple males and mixed eggs from multiple females of the S_V_ morph collected in a rocky area, some offspring presented the S_B_ color phenotype (Kokita et al., unpublished data). This phenomenon strongly suggests that some S_V_ individuals have heterozygous genotypes (a dominant S_V_ allele and a recessive S_B_ allele) for the causal gene underlying color divergence, considering the genetic inheritance pattern of body color as mentioned above. Although the scope of this study did not include discerning intraspecific genetic polymorphism or incipient speciation, we assert that the maintenance of genetically determined color morphs within a homogenous population is likely for this system.

In any case, it is possible that the only gene specifically associated with body color differs between the two color morphs. Our GWAS actually detected that a single SNP on RAD locus 29,926 was strongly associated with the phenotype of each morph. Because RAD locus 29,926 did not show complete genotype–phenotype association based on a single‐locus, two‐allele Mendelian inheritance pattern, with the S_B_ morph having the recessive allele (see Figure 5b), this result does not indicate that this SNP lies within a region in complete linkage disequilibrium of the causal locus underlying phenotypic variation between these morphs. However, there is a strong possibility that this SNP will exist near a locus potentially associated with the color divergence. Because of the genome‐wide synteny between cyprinid fish species belonging to the subfamily Gobioninae and zebrafish (Kakioka et al., 2013), we performed a BLAST search of the two RAD consensus sequences (29,926 and 14,635; see Figure S3) containing significant SNPs against the zebrafish reference genome (GRCz11). However, the highest *E*‐value of the BLAST hit sequences was 0.13; therefore, we were unable to find any homologous regions or syntenic regions. We are currently conducting *de novo* whole‐genome sequencing and assembly, quantitative trait locus (QTL) mapping using the BC cross created in this study, comparative transcriptome analysis using RNA‐Seq from scale skin tissues, and GWAS analysis using whole‐genome resequencing data of wild‐caught individuals. These genetic and genomic studies are likely to identify the actual causal gene and mutation for the loss of dorsoventral countershading in the S_B_ morph in the near future. Further population genetic analyses using the causal gene or its mutation will provide insight into the state along the speciation continuum of this *Sarcocheilichthys* system.

Whether the *Sarcocheilichthys* system examined in this study represents intraspecific polymorphism or two incipient species associated with some degree with premating isolation, this system can be a potentially good model for investigating the functional significance of countershading and the eco‐evolutionary drivers that cause its loss in the aquatic world, where these such studies are rare. As mentioned above, the melanistic morph is a rock‐dwelling specialist that inhabits darker substrates; conversely, the countershaded morph utilizes a wide variety of bottom environments including sandy and pebbly zones with lighter substrates (Komiya et al., 2011). Therefore, the previous study speculated that the uniform dark coloration of the melanistic morph acts as camouflage, albeit briefly, in poorly lit rocky environments (Hosoya, 1982; Komiya et al., 2011). Certainly, since variation in countershading is dependent on the light environment, a dark average tone has often been associated with dark, closed environments (e.g., Allen et al., 2012; Caro et al., 2011; Cuthill et al., 2017). Most such studies have focused on camouflage as an antipredator adaptation (Cuthill, 2019; Cuthill et al., 2017), and the melanistic morph of *Sarcocheilichthys* may also have evolved to prevent its detection and/or recognition by predators. The littoral areas of Lake Biwa contain native piscivorous fishes such as the rock‐dwelling catfish *Silurus lithophilus* and piscivorous chub *Opsariichthys uncirostris* (Okuda et al., 2014), as well as the carnivorous soft shell turtle *Pelodiscus sinensis* (Matsui, 2012; Takigawa et al., 2020); these aquatic animals are potential predators of *Sarcocheilichthys*. Because *Sarcocheilichthys* fish usually swim biased to the subbenthic column (Nakamura, 1969), they face predation threats from the side and above. The complicated bottom environments of the littoral regions of Lake Biwa, including sandy, pebbly, and rocky area, were established about 0.4 million years ago (Meyers et al., 1993). Thus, uniformly dark coloration may offer an evolutionary advantage through matching the visual background in poorly lit, closed rocky environments. Conversely, according to the primary explanation for the evolution of countershading, the countershaded morph may attain concealment by reducing shadows (or background matching) in well‐lit environments like sandy and pebbly areas with lighter substrate. Therefore, the melanistic morph appears to have a cryptic disadvantage in these areas.

However, this scenario cannot explain the distributional patterns of the countershading morph that utilizes a wide variety of bottom environments, especially the rocky environments. The two morphs co‐occur in rocky areas, showing spatial variation in their frequencies, with the melanistic morph representing 10–30% of the total population in these areas. (Nakamura, 1969). Our qualitative observations using SCUBA equipment in 2015 and 2019 confirmed that these color morphs certainly occur together in multiple rocky areas near two localities (codes 7 and 13; Komiya et al., 2014) (Kokita et al., unpublished data). However, although these color morphs co‐occur, they may utilize different microhabitats within rocky areas. Rocky areas in the littoral zone in Lake Biwa exhibit great structural and environmental complexity, and there are many vacant, sandy spaces among the rocks, creating patchy, well‐lit microhabitats. These contrasting microhabitats within the rocky zone may influence the relative fitness of each morph. Gene flow can also play an important role in maintaining polymorphisms between populations, regardless of selective processes acting within population (e.g., Bittner & King, 2003; Saccheri et al., 2008). Komiya et al. (2014) suggested the extensive gene flow among local populations of the countershaded morph inhabiting different bottom environments in Lake Biwa. If this phenomenon represents intraspecific genetic color polymorphism within a homogenous population, several different and complex mechanisms may contribute to maintenance of this stable polymorphism (see Gray & McKinnon, 2007). At this time, we forgo a detailed discussion because it is just speculation.

In conclusion, the results of this study confirmed the coexistence of genetically determined color morphs with and without countershading in the *Sarcocheilichthys* system of Lake Biwa. This color divergence is likely controlled by a single locus, with melanistic color as the recessive alleles. In general, hypotheses explaining the evolution of color polymorphism are often based on the assumption that color morphs are genetically determined (Gray & McKinnon, 2007; White & Kemp, 2016). Therefore, this novel system provides an ideal opportunity for testing hypotheses about the functional aspects of the countershading and its loss in aquatic environments, and for understanding the mechanisms underlying the evolutionary maintenance of color polymorphism in the spatially heterogeneous littoral environments of this lake. Further studies of this system, integrating ecological, genetic, and genomic analyses are needed.

## CONFLICT OF INTEREST

The authors declare that they have no conflicts of interest.

## AUTHOR CONTRIBUTIONS

**Tomoyuki Kokita:** Conceptualization (lead); Data curation (lead); Formal analysis (supporting); Funding acquisition (supporting); Investigation (supporting); Methodology (lead); Project administration (lead); Supervision (lead); Visualization (lead); Writing‐original draft (lead); Writing‐review & editing (lead). **Kohtaro Ueno:** Investigation (lead); Resources (equal). **Yo Y. Yamasaki:** Formal analysis (lead); Writing‐original draft (supporting). **Masanari Matsuda:** Resources (equal); Visualization (supporting). **Ryoichi Tabata:** Resources (equal); Visualization (supporting). **Atsushi J. Nagano:** Investigation (supporting); Resources (supporting). **Tappei Mishina:** Conceptualization (supporting); Formal analysis (supporting). **Katsutoshi Watanabe:** Conceptualization (supporting); Funding acquisition (lead); Project administration (supporting); Resources (equal); Writing‐original draft (supporting); Writing‐review & editing (supporting).

## Supporting information

Figure S1‐S3

## Data Availability

Data about chromatophore analyses are available at https://doi.org/10.5061/dryad.c2fqz618m. All sequence data of the partial coding region of melanin‐related genes (TYR and DCT) and internal control gene (EF1α) for the RT‐qPCR analysis were deposited in DNA Data Bank of Japan (DDBJ) (LC645074–LC645079). The RAD‐seq sequence data are available in DDBJ Sequence Read Archive (DRA) (DRA accession: DRA012383).

## References

[ece38050-bib-0001] Alexander, D. H. , Novembre, J. , & Lange, K. (2009). Fast model‐based estimation of ancestry in unrelated individuals. Genome Research, 19, 1655–1664. 10.1101/gr.094052.109 19648217 PMC2752134

[ece38050-bib-0002] Allen, W. L. , Baddeley, R. , Cuthill, I. C. , & Scott‐Samuel, N. E. (2012). A quantitative test of the predicted relationship between countershading and lighting environment. The American Naturalist, 180, 762–776. 10.1086/668011 23149401

[ece38050-bib-0003] Bittner, T. D. , & King, R. B. (2003). Gene flow and melanism in garter snakes revisited: A comparison of molecular markers and island vs. coalescent models. Biological Journal of the Linnean Society, 79, 389–399. 10.1046/j.1095-8312.2003.00199.x

[ece38050-bib-0004] Braasch, I. , Schartl, M. , & Volff, J. N. (2007). Evolution of pigment synthesis pathways by gene and genome duplication in fish. BMC Evolutionary Biology, 7, 74. 10.1186/1471-2148-7-74 17498288 PMC1890551

[ece38050-bib-0005] Brown, C. M. , Henderson, D. M. , Vinther, J. , Fletcher, I. , Sistiaga, A. , Herrera, J. , & Summons, R. E. (2017). An exceptionally preserved three‐dimensional armored dinosaur reveals insights into coloration and cretaceous predator‐prey dynamics. Current Biology, 27, 2514–2521. 10.1016/j.cub.2017.06.071 28781051

[ece38050-bib-0006] Cal, L. , Suarez‐Bregua, P. , Cerdá‐Reverter, J. M. , Braasch, I. , & Rotllant, J. (2017). Fish pigmentation and the melanocortin system. Comparative Biochemistry and Physiology Part A: Molecular & Integrative Physiology, 211, 26–33. 10.1016/j.cbpa.2017.06.001 28599948

[ece38050-bib-0007] Cal, L. , Suarez‐Bregua, P. , Comesaña, P. , Owen, J. , Braasch, I. , Kelsh, R. , Cerdá‐Reverter, J. M. , & Rotllant, J. (2019). Countershading in zebrafish results from an Asip1 controlled dorsoventral gradient of pigment cell differentiation. Scientific Reports, 9, 3449. 10.1038/s41598-019-40251-z 30837630 PMC6401153

[ece38050-bib-0008] Caro, T. (2005). The adaptive significance of coloration in mammals. BioScience, 55(2), 125–136. 10.1641/0006-3568(2005)055[0125:TASOCI]2.0.CO;2

[ece38050-bib-0009] Caro, T. , Beeman, K. , Stankowich, T. , & Whitehead, H. (2011). The functional significance of colouration in cetaceans. Evolutionary Ecology, 25, 1231–1245. 10.1007/s10682-011-9479-5

[ece38050-bib-0010] Chen, S. , Zhou, Y. , Chen, Y. , & Gu, J. (2018). fastp: An ultra‐fast all‐in‐one FASTQ preprocessor. Bioinformatics, 34, i884–i890. 10.1093/bioinformatics/bty560 30423086 PMC6129281

[ece38050-bib-0011] Cuthill, I. C. (2019). Camouflage. Journal of Zoology, 308, 75–92. 10.1111/jzo.12682

[ece38050-bib-0012] Cuthill, I. C. , Allen, W. L. , Arbuckle, K. , Caspers, B. , Chaplin, G. , Hauber, M. E. , Hill, G. E. , Jablonski, N. G. , Jiggins, C. D. , Kelber, A. , Mappes, J. , Marshall, J. , Merrill, R. , Osorio, D. , Prum, R. , Roberts, N. W. , Roulin, A. , Rowland, H. M. , Sherratt, T. N. , … Caro, T. (2017). The biology of color. Science, 357, eaan0221. 10.1126/science.aan0221 28774901

[ece38050-bib-0013] d'Ischia, M. , Wakamatsu, K. , Cicoira, F. , Di Mauro, E. , Garcia‐Borron, J. C. , Commo, S. , Galván, I. , Ghanem, G. , Kenzo, K. , Meredith, P. , Pezzella, A. , Santato, C. , Sarna, T. , Simon, J. D. , Zecca, L. , Zucca, F. A. , Napolitano, A. , & Ito, S. (2015). Melanins and melanogenesis: From pigment cells to human health and technological applications. Pigment Cell Melanoma Research, 28, 520–544. 10.1111/pcmr.12393 26176788

[ece38050-bib-0014] Gray, S. M. , & McKinnon, J. S. (2007). Linking color polymorphism maintenance and speciation. Trends in Ecology & Evolution, 22, 71–79. 10.1016/j.tree.2006.10.005 17055107

[ece38050-bib-0015] Hendry, A. P. , Bolnick, D. I. , Berner, D. , & Peichel, C. L. (2009). Along the speciation continuum in sticklebacks. Journal of Fish Biology, 75, 2000–2036. 10.1111/j.1095-8649.2009.02419.x 20738669

[ece38050-bib-0016] Hirata, M. , Nakamura, K. I. , & Kondo, S. (2005). Pigment cell distributions in different tissues of the zebrafish, with special reference to the striped pigment pattern. Developmental Dynamics, 234, 293–300. 10.1002/dvdy.20513 16110504

[ece38050-bib-0017] Hochkirch, A. , Deppermann, J. , & Gröning, J. (2008). Phenotypic plasticity in insects: The effects of substrate color on the coloration of two ground‐hopper species. Evolution & Development, 10, 350–359. 10.1111/j.1525-142X.2008.00243.x 18460096

[ece38050-bib-0018] Hosoya, K. (1982). Classification of the cyprinid genus *Sarcocheilichthys* from Japan, with description of a new species. Japanese Journal of Ichthyology, 29, 127–138. 10.11369/jji1950.29.127

[ece38050-bib-0019] Irion, U. , & Nüsslein‐Volhard, C. (2019). The identification of genes involved in the evolution of color patterns in fish. Current Opinion in Genetics & Development, 57, 31–38. 10.1016/j.gde.2019.07.002 31421397 PMC6838669

[ece38050-bib-0020] Janssen, K. , & Mundy, N. I. (2017). The genetic basis and enigmatic origin of melanic polymorphism in pomarine skuas (*Stercorarius pomarinus*). Proceedings of the Royal Society B: Biological Sciences, 284, 20171735. 10.1098/rspb.2017.1735 PMC5740274 29187628

[ece38050-bib-0021] Jombart, T. (2008). adegenet: A R package for the multivariate analysis of genetic markers. Bioinformatics, 24, 1403–1405. 10.1093/bioinformatics/btn129 18397895

[ece38050-bib-0022] Jombart, T. , & Ahmed, I. (2011). adegenet 1.3‐1: New tools for the analysis of genome‐wide SNP data. Bioinformatics, 27, 3070–3071. 10.1093/bioinformatics/btr521 21926124 PMC3198581

[ece38050-bib-0023] Kakioka, R. , Kokita, T. , Kumada, H. , Watanabe, K. , & Okuda, N. (2013). A RAD‐based linkage map and comparative genomics in the gudgeons (genus *Gnathopogon*, Cyprinidae). BMC Genomics, 14, 32. 10.1186/1471-2164-14-32 23324215 PMC3583795

[ece38050-bib-0024] Kawabe, T. (1994). Biwako no Oitachi (formation of Lake Biwa). In Research Group for Natural History of Lake Biwa (Ed.), Biwako no Shizenshi (The natural history of Lake Biwa) (pp. 24–72). Yasaka Shobo.

[ece38050-bib-0025] Kelley, J. L. , & Merilaita, S. (2015). Testing the role of background matching and self‐shadow concealment in explaining countershading coloration in wild‐caught rainbowfish. Biological Journal of the Linnean Society, 114, 915–928. 10.1111/bij.12451

[ece38050-bib-0026] Kelley, J. L. , Taylor, I. , Hart, N. S. , & Partridge, J. C. (2017). Aquatic prey use countershading camouflage to match the visual background. Behavioral Ecology, 28, 1314–1322. 10.1093/beheco/arx093

[ece38050-bib-0027] Kiltie, R. A. (1988). Countershading: Universally deceptive or deceptively universal? Trends in Ecology & Evolution, 3(1), 21–23. 10.1016/0169-5347(88)90079-1 21227055

[ece38050-bib-0028] Kingsley, E. P. , Manceau, M. , Wiley, C. D. , & Hoekstra, H. E. (2009). Melanism in *Peromyscus* is caused by independent mutations in Agouti. PLoS One, 4, e6435. 10.1371/journal.pone.0006435 19649329 PMC2713407

[ece38050-bib-0029] Komiya, T. , Fujita, S. , & Watanabe, K. (2011). A novel resource polymorphism in fish, driven by differential bottom environments: An example from an ancient lake in Japan. PLoS One, 6, e17430. 10.1371/journal.pone.0017430 21387005 PMC3046152

[ece38050-bib-0030] Komiya, T. , Fujita‐Yanagibayashi, S. , & Watanabe, K. (2014). Multiple colonizations of Lake Biwa by *Sarcocheilichthys* fishes and their population history. Environmental Biology of Fishes, 97, 741–755. 10.1007/s10641-013-0176-9

[ece38050-bib-0031] Kopelman, N. M. , Mayzel, J. , Jakobsson, M. , Rosenberg, N. A. , & Mayrose, I. (2015). CLUMPAK: A program for identifying clustering modes and packaging population structure inferences across *K* . Molecular Ecology Resources, 15, 1179–1191. 10.1111/1755-0998.12387 25684545 PMC4534335

[ece38050-bib-0032] Lindgren, J. , Sjövall, P. , Carney, R. M. , Uvdal, P. , Gren, J. A. , Dyke, G. , Schultz, B. P. , Shawkey, M. D. , Barnes, K. R. , & Polcyn, M. J. (2014). Skin pigmentation provides evidence of convergent melanism in extinct marine reptiles. Nature, 506, 484–488. 10.1038/nature12899 24402224

[ece38050-bib-0033] Livak, K. J. , & Schmittgen, T. D. (2001). Analysis of relative gene expression data using real‐time quantitative PCR and the 2^−^ ^ΔΔC^ _T_ method. Methods, 25, 402–408. 10.1006/meth.2001.1262 11846609

[ece38050-bib-0034] Matsuda, N. , Kasagi, S. , Nakamaru, T. , Masuda, R. , Takahashi, A. , & Tagawa, M. (2018). Left‐right pigmentation pattern of Japanese flounder corresponds to expression levels of melanocortin receptors (MC1R and MC5R), but not to agouti signaling protein 1 (ASIP1) expression. General and Comparative Endocrinology, 262, 90–98. 10.1016/j.ygcen.2018.03.019 29574149

[ece38050-bib-0035] Matsui, M. (2012). Amphibians and reptiles in and around Lake Biwa. In H. Kawanabe , M. Nishino , & M. Maehata (Eds.), Lake Biwa: Interactions between nature and people (pp. 123–128). Springer.

[ece38050-bib-0036] McCurley, A. T. , & Callard, G. V. (2008). Characterization of housekeeping genes in zebrafish: Male‐female differences and effects of tissue type, developmental stage and chemical treatment. BMC Molecular Biology, 9, 102. 10.1186/1471-2199-9-102 19014500 PMC2588455

[ece38050-bib-0037] McKinnon, J. S. , & Pierotti, M. E. (2010). Colour polymorphism and correlated characters: Genetic mechanisms and evolution. Molecular Ecology, 19, 5101–5125. 10.1111/j.1365-294X.2010.04846.x 21040047

[ece38050-bib-0038] McRobie, H. R. , Moncrief, N. D. , & Mundy, N. I. (2019). Multiple origins of melanism in two species of North American tree squirrel (*Sciurus*). BMC Evolutionary Biology, 19, 140. 10.1186/s12862-019-1471-7 31296164 PMC6625063

[ece38050-bib-0039] Meirmans, P. G. (2020). GENODIVE version 3.0: Easy‐to‐use software for the analysis of genetic data of diploids and polyploids. Molecular Ecology Resources, 20, 1126–1131. 10.1111/1755-0998.13145 32061017 PMC7496249

[ece38050-bib-0041] Meyers, P. A. , Takemura, K. , & Horie, S. (1993). Reinterpretation of late Quaternary sediment chronology of Lake Biwa, Japan, from correlation with marine glacial‐interglacial cycles. Quaternary Research, 39, 154–162. 10.1006/qres.1993.1019

[ece38050-bib-0042] Nachman, M. W. (2005). The genetic basis of adaptation: Lessons from concealing coloration in pocket mice. Genetica, 123, 125–136. 10.1007/s10709-004-2723-y 15881685

[ece38050-bib-0043] Nachman, M. W. , Hoekstra, H. E. , & D'Agostino, S. L. (2003). The genetic basis of adaptive melanism in pocket mice. Proceedings of the National Academy of Sciences of the United States of America, 100, 5268–5273. 10.1073/pnas.0431157100 12704245 PMC154334

[ece38050-bib-0044] Nakamura, M. (1969). Cyprinid fishes of Japan: Studies on the life history of cyprinid fishes of Japan. Special Publications of the Research Institute for Natural Resources, Tokyo, 4, 1–455.

[ece38050-bib-0045] Nosil, P. (2012). Ecological speciation. Oxford University Press.

[ece38050-bib-0046] Okuda, N. , Watanabe, K. , Fukumori, K. , Nakano, S. , & Nakazawa, T. (Eds.). (2014). Origin and diversification of freshwater fishes in Lake Biwa. Biodiversity in aquatic systems and environments. SpringerBriefs in Biology (pp. 1–19). Springer. 10.1007/978-4-431-54150-9_1

[ece38050-bib-0047] Orteu, A. , & Jiggins, C. D. (2020). The genomics of coloration provides insights into adaptive evolution. Nature Reviews Genetics, 21, 461–475. 10.1038/s41576-020-0234-z 32382123

[ece38050-bib-0048] Peterson, B. K. , Weber, J. N. , Kay, E. H. , Fisher, H. S. , & Hoekstra, H. E. (2012). Double digest RADseq: An inexpensive method for de novo SNP discovery and genotyping in model and non‐model species. PLoS One, 7, e37135. 10.1371/journal.pone.0037135 22675423 PMC3365034

[ece38050-bib-0049] Potash, A. D. , Greene, D. U. , Mathis, V. L. , Baiser, B. , Conner, L. M. , & McCleery, R. A. (2020). Ecological drivers of eastern fox squirrel pelage polymorphism. Frontiers in Ecology and Evolution, 8, 119. 10.3389/fevo.2020.00119

[ece38050-bib-0050] R Core Team (2020). R: A language and environment for statistical computing. R Foundation for Statistical Computing. https://www.R‐project.org/

[ece38050-bib-0051] Raymond, M. , & Rousset, F. (1995). GENEPOP (Version 1.2): Population genetics software for exact tests and ecumenicism. Journal of Heredity, 86, 248–249. 10.1093/oxfordjournals.jhered.a111573

[ece38050-bib-0052] Rochette, N. C. , Rivera‐Colón, A. G. , & Catchen, J. M. (2019). Stacks 2: Analytical methods for paired‐end sequencing improve RADseq‐based population genomics. Molecular Ecology, 28, 4737–4754. 10.1111/mec.15253 31550391

[ece38050-bib-0053] Roulin, A. (2014). Melanin‐based colour polymorphism responding to climate change. Global Change Biology, 20, 3344–3350. 10.1111/gcb.12594 24700793

[ece38050-bib-0054] Rowland, H. M. (2009). From Abbott Thayer to the present day: What have we learned about the function of countershading? Philosophical Transactions of the Royal Society B: Biological Sciences, 364, 519–527. 10.1098/rstb.2008.0261 PMC2674085 19000972

[ece38050-bib-0055] Ruxton, G. D. , Speed, M. P. , & Kelly, D. J. (2004). What, if anything, is the adaptive function of countershading? Animal Behaviour, 68, 445–451. 10.1016/j.anbehav.2003.12.009

[ece38050-bib-0056] Saccheri, I. J. , Rousset, F. , Watts, P. C. , Brakefield, P. M. , & Cook, L. M. (2008). Selection and gene flow on a diminishing cline of melanic peppered moths. Proceedings of the National Academy of Sciences of the United States of America, 105, 16212–16217. 10.1073/pnas.0803785105 18854412 PMC2571026

[ece38050-bib-0057] Sakaguchi, S. , Sugino, T. , Tsumura, Y. , Ito, M. , Crisp, M. D. , Bowman, D. M. J. S. , Nagano, A. J. , Honjo, M. N. , Yasugi, M. , Kudoh, H. , Matsuki, Y. , Suyama, Y. , & Isagi, Y. (2015). High‐throughput linkage mapping of Australian white cypress pine (*Callitris glaucophylla*) and map transferability to related species. Tree Genetics & Genomes, 11, 121. 10.1007/s11295-015-0944-0

[ece38050-bib-0058] Tabata, R. , Kakioka, R. , Tominaga, K. , Komiya, T. , & Watanabe, K. (2016). Phylogeny and historical demography of endemic fishes in Lake Biwa: The ancient lake as a promoter of evolution and diversification of freshwater fishes in western Japan. Ecology and Evolution, 6, 2601–2623. 10.1002/ece3.2070 27066244 PMC4798153

[ece38050-bib-0059] Takigawa, Y. , Kato, S. , Nakano, T. , Nakai, K. , Tomikawa, K. , Ishiwata, S. , Fujita, T. , Hosoya, K. , Kawase, S. , Senou, H. , Yoshino, T. , & Nishino, M. (2020). The Vega collection at the end of the nineteenth‐century survey of Lake Biwa. In H. Kawanabe , M. Nishino , & M. Maehata (Eds.), Lake Biwa: Interactions between nature and people (2nd ed., pp. 247–258). Springer.

[ece38050-bib-0060] Thayer, A. H. (1896). The law which underlies protective coloration. The Auk, 13, 124–129. 10.2307/4068693

[ece38050-bib-0061] Venables, S. K. , Marshall, A. D. , Germanov, E. S. , Perryman, R. J. Y. , Tapilatu, R. F. , Hendrawan, I. G. , Flam, A. L. , van Keulen, M. , Tomkins, J. L. , & Kennington, W. J. (2019). It’s not all black and white: Investigating colour polymorphism in manta rays across Indo‐Pacific populations. Proceedings of the Royal Society B: Biological Sciences, 286, 20191879. 10.1098/rspb.2019.1879 PMC6790782 31594509

[ece38050-bib-0062] Vidal, O. , Araguas, R. M. , Fernández, R. , Heras, S. , Sanz, N. , & Pla, C. (2010). Melanism in guinea fowl (*Numida meleagris*) is associated with a deletion of Phenylalanine‐256 in the MC1R gene. Animal Genetics, 41, 656–658. 10.1111/j.1365-2052.2010.02056.x 20477788

[ece38050-bib-0063] White, T. E. , & Kemp, D. J. (2016). Colour polymorphism. Current Biology, 26, R517–R518. 10.1016/j.cub.2016.03.017 27404233

[ece38050-bib-0064] Yokoyama, T. (1984). Stratigraphy of the quaternary system around Lake Biwa and geohistory of the ancient Lake Biwa. In S. Horie (Ed.), Lake Biwa, monographiae biologicae (Vol. 54, pp. 43–128). Dr. W. Junk Publishers.

[ece38050-bib-0065] Zhou, X. , & Stephens, M. (2012). Genome‐wide efficient mixed‐model analysis for association studies. Nature Genetics, 44, 821–824. 10.1038/ng.2310 22706312 PMC3386377

