# Supplementary material for: Gudgeon fish with and without genetically determined countershading coexist in heterogeneous littoral environments of an ancient lake
Source: Ecol Evol. 2021 Aug 31;11(19):13283–94. doi: 10.1002/ece3.8050 (PMC8495823; doi:10.1002/ece3.8050)
Supplement: Supplementary file 1 — Figure S1‐S3 [file ECE3-11-13283-s001.docx]

**Appendix**


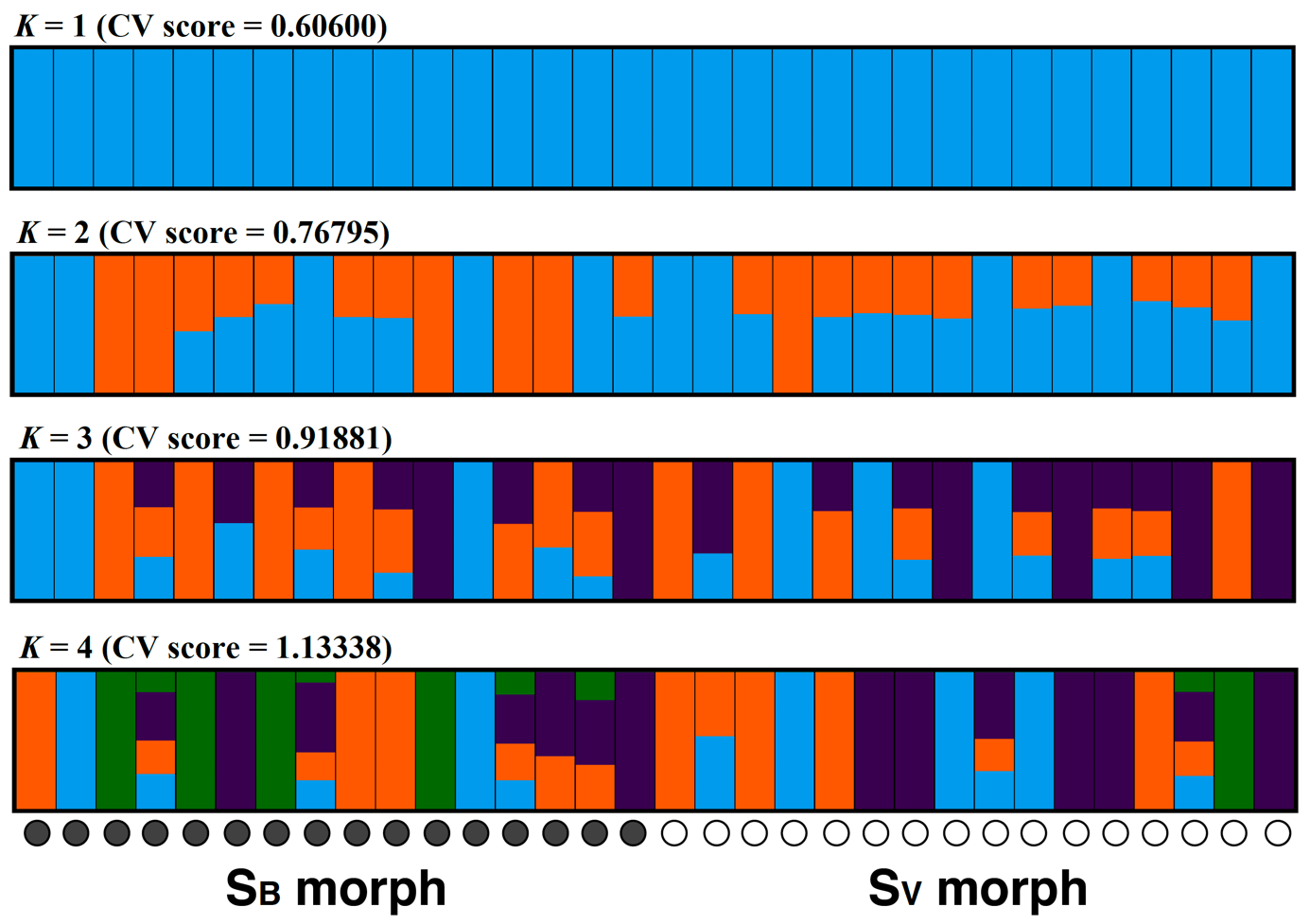


**FIGURE S1** Barplots representing individual genetic cluster assignment from ADMIXTURE results from *K* = 1 to *K* = 4. Cross validation (CV) scores are shown for each *K* value.

**
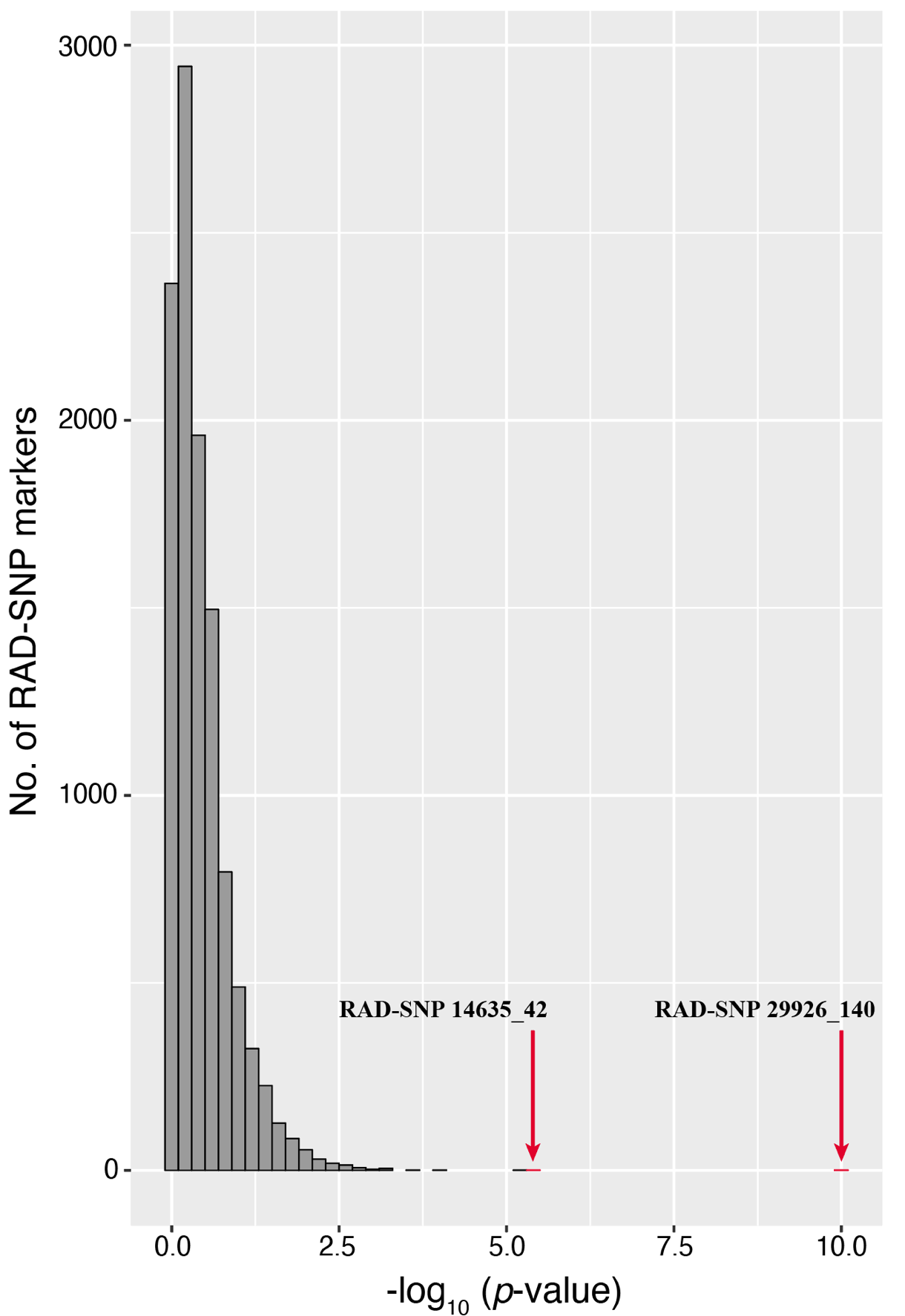
**

**FIGURE S2** The result of the genome-wide association analysis of color phenotype using the method genome-wide efficient mixed-model association (GEMMA). Two RAD-SNPs, 29926_140 and 14635_42, showed a statistically significant association (*p* < 0.05 after Bonferroni correction).

**RAD locus_29926**

ATTCCGCCAAGTATATCAGCATCGTCATACCTGTTACCATGCCAAACTGCCCGAGAGTTGTCATGACAGAAATGCCTATTTAGGTATTGTTTTAGCAAACTTTTCACAATTCCAGATGATAAAATAAAGTCTCGCTCAG[C/T]ATTTTTCTGTTGAATGTTTCTCTTATAAATGCAAACAAATGGCAACTTTAG

**RAD locus _14635**

CTCTCACCAGTTGTTTTCAGTGAAATAAATGCATTGATCTG[A/G]GAAGTGGTTCACCATCTGAGTAAGTGTTTGCTGTCATCTGAATAAGCTGTTTCATGGACNNNNNNNNNNAGATATAACATATGATATGAATATGATAATTATCCGTATGGTATTTTTATAGAACCATTTGATACCAAAACCAGTTTGTGGCACCACAGTACCTTTCTTTAG

**FIGURE S3** Two RAD consensus sequences containing a significant SNP (29926_140 and 14635_42; highlighted in yellow).
